# Preventive strategies for recurrent urinary tract infections in premenopausal women: A scoping review

**DOI:** 10.1080/13814788.2026.2661165

**Published:** 2026-05-14

**Authors:** Caroline Skovsbo Clausen, Louise Bidstrup Jørgensen, Rune Aabenhus, Sif Helene Arnold, Katrine Hartung Hansen, Lars Bjerrum, Mette Bech Risør, Anne Holm

**Affiliations:** a Department of Public Health, Center for General Practice, University of Copenhagen, København K, Denmark; b Department of Microbiology, Hospital of Region Zealand, Køge, Denmark

**Keywords:** rUTI, antimicrobial resistance, preventive strategies for rUTIs, evidence gaps, clinical guidance and shared decision-making

## Abstract

**Background:**

Recurrent urinary tract infection (rUTI) in women is common in primary care. Antibiotics can prevent rUTI, but their use is associated with the risk of developing antimicrobial resistance. Thus, non-antibiotic strategies are needed, yet evidence remains limited due to methodologically weak studies. Consequently, potentially effective strategies lack sufficient evidence for guideline inclusion, leaving healthcare professionals (HCPs) and women with little support. This scoping review synthesises preventive strategies for rUTIs in premenopausal women, mapping recommendations and underlying rationales to guide future research and guideline development.

**Methods:**

This Scoping review was conducted by searching PubMed, Cochrane, EMBASE, Web of Science, and CINAHL. 6170 records were identified for eligibility. A total of 78 publications were included, i.e. reviews, guidelines, and expert reports regarding preventive strategies used for rUTIs for premenopausal women, published between January 2013 and December 2023.

**Results:**

78 publications addressed rUTIs prevention in premenopausal women, encompassing both well-studied strategies, such as antibiotic regimens and cranberry products, and less-studied approaches, including behavioural modifications, vitamin supplements, and increased hydration. Recommendations were often unsupported by evidence, and disagreement among sources was common.

**Discussion:**

To support women with rUTIs, HCPs require an overview of preventive strategies including supporting recommendations and evidence. Our findings highlight a clear contrast between well-studied strategies, such as antibiotic regimens, and behavioural strategies, where evidence is limited, often because some cannot feasibly be evaluated in randomised trials rather than due to limited clinical relevance. These findings may help HCPs and guideline developers assess evidence and prioritise future research.

## KEY MESSAGES

1. Recurrent urinary tract infections are a major challenge in primary care, affecting patient well-being and healthcare resources.
2. Preventive strategies are diverse, but evidence is limited and inconsistent, leaving GPs uncertain and highlighting the need for shared decision-making.
3. Mapping existing strategies and evidence gaps provides a foundation for future research and clearer clinical guidelines.

## Introduction

Urinary tract infections (UTIs) represent one of the most prevalent bacterial infections in primary care, with an estimated global annual incidence of 150 million cases [1,2]. It is estimated that up to 50% of women will experience a UTI at some point in their lives, with 20-30% of these individuals experiencing recurrent UTIs (rUTIs) [3,4]. rUTIs are defined by the occurrence of ≥2 infections in 6 months or ≥3 infections in 12 months [4].

Typical symptoms of UTIs are bothersome [5]. Women with rUTIs report that these symptoms significantly lower their overall quality of life, affecting both sexual and social relationships, self-esteem, and work capacity [3,5–7]. Consequently, prevention remains a pressing challenge for HCPs and patients alike.

rUTIs can be partly prevented with antibiotics, but this strategy involves unwanted effects, such as the risk of promoting side effects and the risk of resistant bacteria [8,9]. Pursuing non-antibiotic strategies to prevent rUTIs aligns with the World Health Organization’s (WHO’s) designation of antimicrobial resistance (AMR) as a major threat to global public health [10]. An increased focus on alternatives to antibiotics in the prevention of rUTIs has become essential over the past decade [11,12]. Numerous studies have investigated both antibiotic and non-antibiotic preventive strategies for rUTIs. However, the evidence supporting the efficacy of non-antibiotic strategies is often not robust due to limitations in study design or execution of the study [13]. While some strategies, such as cranberries, have been extensively researched [14–16], other strategies, frequently used by women or recommended by clinicians, such as post‑coital voiding, limiting the use of spermicides, increased hydration or wearing cotton underwear, have never, or only to a very limited extent, been subject to clinical trials [17,18]. This leads to a knowledge gap, where potentially effective strategies cannot be strongly recommended in guidelines due to a lack of evidence [17]. Potential reasons for this unequal evidence base could be a general neglect of conditions predominantly affecting women [19].

Existing studies on women’s perspectives indicate a need for improved management of rUTIs through research with greater emphasis on prevention, development of non-antibiotic treatment options, and support for patient self-management, alongside enhanced antibiotic stewardship [20,21].

A scoping review is particularly well-suited for this purpose, as it enables a comprehensive mapping of currently recommended strategies while also exploring the underlying mechanisms behind these recommendations. This is desirable in a field where robust evidence is lacking, and can help inform future research and guideline development.

## Objective

The objective of this scoping review was to provide a comprehensive and representative overview of expert recommendations on preventive strategies for rUTIs in premenopausal women based on a systematic search of the scientific literature conducted over 10 years (2013–2023). For each recommendation, we aimed to provide the corresponding rationales provided by experts.

## Methods

### Search strategy

We searched through the databases PubMed, Cochrane, EMBASE, Web of Science, and CINAHL for studies published between 01.01.2013-08.12.2023, written in English, Danish, Swedish, or Norwegian concerning the prevention of rUTI in adult, premenopausal women. To capture a thorough extraction of all existing preventive strategies in the scientific literature, we included any peer-reviewed publication with one of the following designs:

- Reviews, i.e., *expert reviews, scoping reviews, umbrella reviews, literature reviews, narrative reviews, clinical reviews, systematic reviews (studies investigating more than one preventive strategy)*
- Guidelines (updated guidelines and guidelines of guidelines)
- Reports on recommendations formulated by specialty-relevant networks, expert opinion papers

This approach aimed at comprehensively mapping preventive strategies, including those beyond the scope of clinical trial evaluations. Additionally, we sought to examine expert recommendations, which were well represented in the included publications. ‘Experts’ were defined as authors of reviews, guidelines, and other peer-reviewed expert opinion papers who expressed recommendations by addressing or referring to clinical practice and existing research. Guidelines were defined in accordance with the IOM framework for developing trustworthy clinical practice guidelines [22]. The search strategy was formulated in collaboration with an information specialist and through discussions within the research group. The full search strategy can be seen in the *Appendix.* Articles were deduplicated in EndNote and Covidence before screening in Covidence. The identified titles and abstracts were screened for eligibility by two independent reviewers (CSC and SHA). Full texts were screened by two independent reviewers (CSC and SHA or CSC and AH).

The scoping review was conducted in accordance with the Joanna Briggs Institute (JBI) guidelines and reported according to PRISMA Extension for Scoping Reviews (PRISMA-ScR) standards [23–25]. An a priori protocol was uploaded to the Open Science Framework Database in December 2023 and updated in April 2025.

### Data extraction process

A data extraction guidance form was conducted in close relation to the research question. The data extraction form was accompanied by an extraction guidance document, based on the principles advised by Pollock et al. to ensure a consistent extraction within the research group [26].

Two independent reviewers (CSC and AH) extracted descriptive data, which included 1^st^ authors, year of publication, country, and category of study design (Table 1). Any disagreements were resolved through discussion. The data extraction process, including the list of preventive strategies, the categorisation of recommendations, and the rationales behind the recommendations, was carried out by five reviewers in pairs of two (CSC or LBJ and SHA, KHH, LB, MBR, or RAA).

**Table 1.** Description of the 78 included publications. Studies assigned to the category review were those where review were unspecified by the authors.

First author	Year of publication	Country	Category
Abou Heidar, N. [38]	2019	Lebanon	Review
Al-Badr, A. [39]	2013	Saudi Arabia	Review
Anger, J. [30]	2019	United States	Guideline system
Anger, J. T. [40]	2022	United States	Updated Guideline
Aragón, I. M. [41]	2018	Spain	Systematic Review
Arnold, James J. [42]	2016	United States	Clinical review
Aslam, S. [43]	2020	United States	Clinical review
Barber, A. E. [44]	2013	United States	Review
Barclay, J. [45]	2017	United Kingdom	Clinical review
Barea, B. M. [46]	2020	United Kingdom	Review
Beerepoot, M. A. [47]	2016	Netherlands	Review
Beerepoot, M. [48]	2013	Netherlands	Systematic review
Bergamin, P. A. [31]	2017	Australia	Review
Betschart, C. [49]	2020	Switzerland	Guideline
Brubaker, L. [33]	2018	United States	Guideline
Cai, T. [50]	2017	Italy	Review
Caron, F. [51]	2018	France	Guideline
Chen, Y. C. [52]	2023	Taiwan	Review
Chetwood, A. [28]	2014	United Kingdom	Review
Christofides, A. [53]	2013	United Kingdom	Review
Costantini, E. [54]	2017	Italy	Review
da Silva, A. L. [55]	2017	Brazil	Editorial
de Rossi, P. [56]	2020	Brazil	Guideline
Epp, A. [57]	2017	Canada	Guideline
Farford, B. [58]	2018	United States	Review
Feng, Feng [59]	2018	United States	Literature Review
Finney, E. L. [60]	2022	United States	Review
Foxman, B. [61]	2013	United States	Review
Geerlings, S. E. [62]	2014	Netherlands	Review
Glover, E. K. [63]	2023	United Kingdom	Clinical review
Glover, M. [64]	2014	United States	Review
Guglietta, A. [65]	2017	Norway	Review
Gupta, Kalpana [66]	2013	United States	Clinical Review
Haddad, J. M. [67]	2020	Brazil	Review
Harding, C. [68]	2019	United Kingdom	Expert opinion
Hernandez-Hernandez, D. [69]	2022	Spain	Review
Hickling, D. R. [70]	2013	United States	Review
Hutton, H.; Amos, L. [71]	2014	Australia	Clinical review
Jarvis, T. R. [72]	2014	Australia	Clinical review
Jhang, J. F. [73]	2017	Taiwan	Review
Kolman, K. B. [74]	2019	United States	Review
Kranz, J. [75]	2018	Germany	Guideline
Kranz, J. [76]	2019	Germany	Clinical review
Krishnaswamy, P. H. [77]	2020	United Kingdom	Review
Kucheria, A. [78]	2023	United Kingdom	Practical guide
Kwok, M. [17]	2022	Australia	Guideline of Guidelines (review)
Lee, D. S. [79]	2018	South Korea	Review
Liska, D. [80]	2018	United States	Clinical review
Lodhia, S. [81]	2020	United Kingdom	Clinical review
Martell, J. A. O. [82]	2019	Mexico	Expert opinion
Martin, Christy D. [83]	2019	United States	Literature Review
Miranne, J. M. [84]	2017	United States	Review
Moussa, M. [85]	2020	Lebanon	Review
Negus, M. [86]	2020	United Kingdom	Review
Nikpoor, P. [87]	2020	Australia	Clinical review
O’Brien, V. P. [88]	2016	United States	Clinical review
O’Riordan [89]	2023	United Kingdom	Clinical review
Panesar, K. [90]	2013	United States	Clinical review
Peck, J. [29]	2021	United States	Expert review
Scribano, D. [91]	2021	Italy	Opinion review
Shepherd, A. K. [92]	2013	United States	Clinical review
Sihra, N. [11]	2018	United Kingdom	Clinical review
Sihra, N. [93]	2022	United Kingdom	Expert opinion
Silverman, J. A. [94]	2013	United States	Review
Smith, A. L. [32]	2018	United States	Rapid Review
Sosland [95]	2020	United States	Review
Stair, S. L [96]	2023	United States	Review
Taich, L. [97]	2020	United States	Review
Tamadonfar, K. O. [98]	2019	United States	Expert opinion
Vahlensieck, W. [99]	2016	Germany	Clinical review
Van Wietmarschen [100]	2022	Netherlands	Literature Review
Vecchio [101]	2018	France	Review
Vedanayagam, M. [102]	2013	United Kingdom	Literature Review
Wagenlehner, F. [103]	2021	Germany	Expert consensus
Wasson. [104]	2015	United States	Review
Yang, B. [105]	2019	United Kingdom	Review
Zak, D. [106]	2014	United States	Expert opinion
Zare, M. [107]	2022	Germany	Narrative Review

Before commencing data extraction, an agreement of at least 80% between the first and second rounds was established as sufficient. Following the double extraction, an agreement exceeding >95% was reached. Conflicts were resolved between CSC, AH, and RAA.

#### Methods used to develop the list of preventive strategies for rUTIs

All preventive strategies for rUTIs in premenopausal women were extracted. Using the principles of qualitative content analysis, a coding framework was developed to categorise and subcategorise these strategies [26,27]. The framework was discussed within the research group (AH, CSC, LB) to ensure alignment with medical classifications and later reviewed by a pharmacology specialist (LB). The final framework comprised eight categories, each with multiple subcategories.

#### Methods used to categorise the recommendations on preventive strategies for rUTIs

The preventive strategies were accompanied by corresponding rationales for the recommendation, which were extracted as text sections. To analyse these rationales, an initial framework was developed to categorise the recommendations. This framework comprised four categories, with corresponding colours: recommended (green), may be recommended (yellow), neutral (white), or not recommended (red) as depicted in Table 2. The recommendation *neutral* encompassed recommendations where the authors did not take a stance on whether the strategy should be recommended or not, or it was directly stated that there was insufficient evidence to make definitive recommendations. An average of these recommendations was calculated by counting the number of colours associated with each strategy. Consequently, colours shown in Table 2 represent the average recommendation for each preventive strategy.

**Table 2.** Preventive strategies used in recurrent urinary tract infections for premenopausal women arranged by eight distinct categories: 1) Pharmaceuticals, 2) Antibiotic regimens, 3) Behavioral modifications, 4) Complementary approaches, 5) Dietary modifications, 6) Dietary supplements, 7) Medical Procedures, and 8) Others, and their respective subcategories. The preventive strategies’ associated recommendations are visualised by colour schemes (green = recommended, yellow = may be recommended, white = neutral, and red = not recommended), and the colours represent an average recommendation. The agreement index measures the degree of consensus among experts’ recommendations in the included publications.

Preventive strateges used for recurrent urinary tract infections	Number of articles (*n*)	Recommendation	Agreement Index (percentage)
**Pharmaceuticals**			** **
Antibiotics (no regimen specified)	16	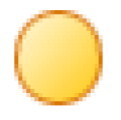	100%
Anti-infective: Methenamine Hippurate	38	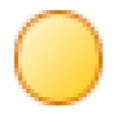	32%
Immunostimulants (vaccines)	49	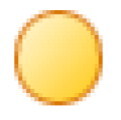	13%
NSAID	3	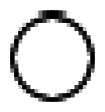	33%
**Antibiotic Regimens**		** **	
Low dose continuous antibiotic prophylaxis	40	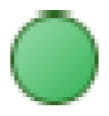	95%
Postcoital antibiotic prophylaxis	33	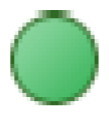	88%
Self-start antibiotics	18	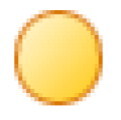	100%
**Behavioural modifications**		** **	
Avoiding anal intercourse	2	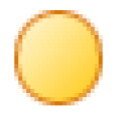	100%
Avoiding hypothermia	2	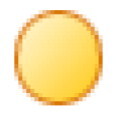	100%
Avoiding intercourse with multiple sexual partners	2	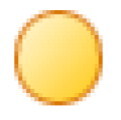	100%
Avoiding obesity	2	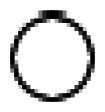	100%
Avoiding postponing urination	13	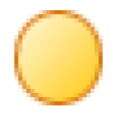	23%
Avoiding sexual intercourse	5	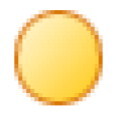	20%
Avoiding skin allergens (soaps, vaginals creams, bubble baths, hot tubs etc.)	12	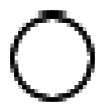	17%
Avoiding spermicide-based contraceptives (diaphragm use, condoms etc.)	8	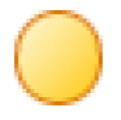	75%
Avoiding tampons/prolonged use of pads	3	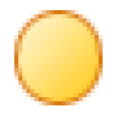	33%
Behavioural modifications (non specified)	20	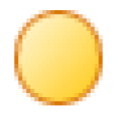	10%
Cleaning genital areas before and after sexual intercourse	1	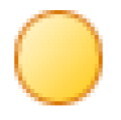	n/a
Complete bladder emptying	5	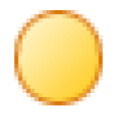	20%
Limiting use of spermicides	17	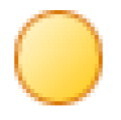	100%
Postcoital-voiding	10	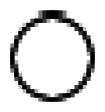	0%
Proper wiping patterns	18	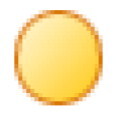	22%
Wear cotton underwear	8	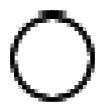	0%
**Complementary Approaches**		** **	
Acupuncture	14	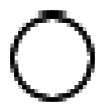	71%
Ayurveda and Unani	1	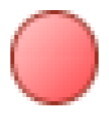	n/a
Chinese herbal medicines	10	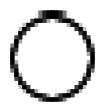	40%
Massage and Touch therapies, reflexology	1	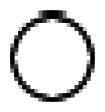	n/a
Phytoterapeutics	14	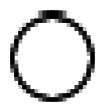	22%
Yoga	1	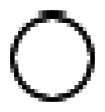	n/a
**Dietary Modifications**		** **	
Increased hydration	24	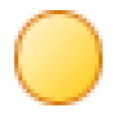	42%
Specific diet	5	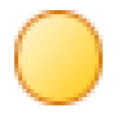	20%
**Dietary Supplements**		** **	
Anti-adhesive therapeutics: Pillicides and mannosides	3	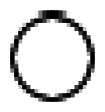	100%
Cranberry products	69	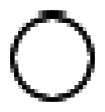	19%
D-mannose	43	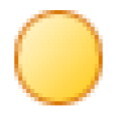	12%
Glycolipids/galactocides	3	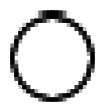	100%
L-arginine	1	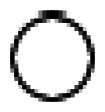	n/a
Other acidifying agents	2	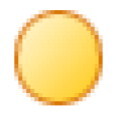	100%
Other alkalising agents	5	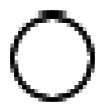	60%
Probiotics (different Lactobacilli)	50	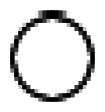	58%
Vitamins (C and D)	13	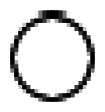	60%
**Medical procedures**		** **	
Faecal transplantation	2	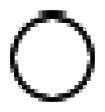	0%
Fractional CO2 laser and distal urethral transposition	1	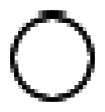	n/a
Intravesical installations	32	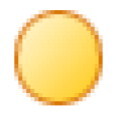	3%
Urethral dilation	5	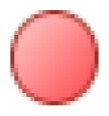	100%
**Others**		** **	
Addressing concomitant faecal incontinence	1	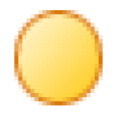	n/a
Bacteriophages	3	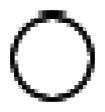	100%
Combination of products	2	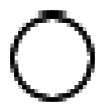	100%
Improving bowel movement	1	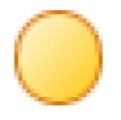	n/a
Intestinal mucosal barrier agents	1	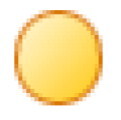	n/a
Topical hyaluronic acid	1	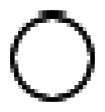	n/a

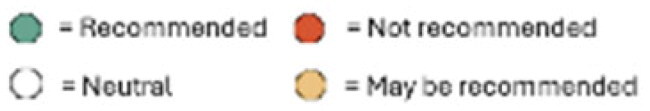

#### Calculating the agreement index

To illustrate the variation in recommendations within each preventive strategy, we calculated an agreement index valued 0–100%. A percentage in which 100% indicated that all recommendations were either fully positive or fully negative, while 0% indicated that the recommendations were evenly distributed. We started by sorting the four possible recommendations as either positive (‘recommended’ or ‘may be recommended’) or negative (‘neutral’ or ‘not recommended’). As an illustration, the agreement index for Methenamine Hippurate was calculated based on a total of 38 recommendations, comprising 25 positive and 13 negative recommendations. The agreement index was derived by subtracting the proportion of negative recommendations from the proportion of positive recommendations, using the following formula: (25/38 × 100%) − (13/38 × 100%), yielding an overall agreement index of 32%.

### Methods used to categorise the rationales for the recommendations on preventive strategies for rUTIs

Adhering to the basic principles of qualitative content analysis, we developed a coding framework during an open coding process to analyse the rationales [26,27]. We aimed to identify the different rationales used for the different recommendations rather than extract the results of evidence sources. This approach aligns with the recommendations for conducting a scoping review as outlined by Pollock and colleagues [26]. The rationales were developed through a highly text-proximate and inductive analytic process, ensuring that the rationale categories closely reflected how the authors of the included studies articulated their own arguments. The coding framework encompassed six different categories of rationales used for the recommendations: *effectiveness, harms*, *mechanisms*, *usefulness*, *common sense*, and *statements.* This coding framework was used to organise the rationales accordingly. As an example, one publication identified limiting the use of spermicides as a potential preventive strategy, which was categorised under Behavioural Modifications. The authors noted that avoiding spermicides may reduce vaginal colonisation associated with nonoxynol-9 and that switching to alternative contraceptives could be beneficial [28]. As the rationale relied on author statements rather than empirical evidence, this strategy was classified as ‘may be recommended’, with the rationale coded as Statement. The categories shown in Figure 3 illustrate the proportional distribution of rationales applied across the preventive strategy categories, expressed as a percentage. Additional details on these rationale categories are provided in Box 1.

## Results

### Search results

**Figure 1.**
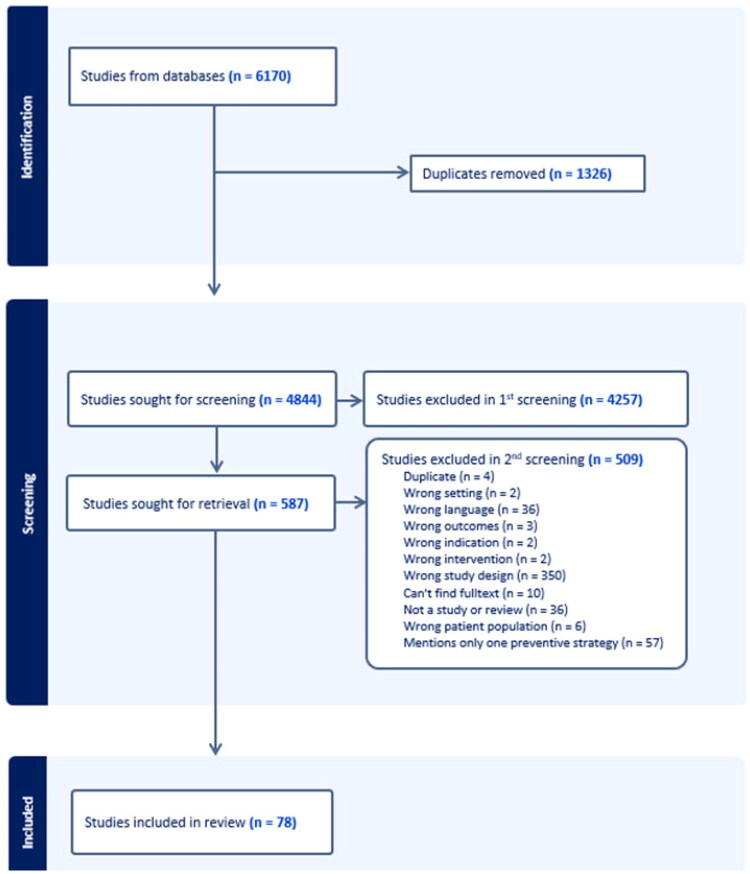
PRISMA flow diagram showing the search strategy used to identify the included publications.

A total of 6170 records were identified. After removing duplicates, 4844 records were screened by title and abstract. Following two rounds of screening, 78 publications were included. The selection process is illustrated in Figure 1 (PRISMA-chart).

### Geographical representation

**Figure 2.**
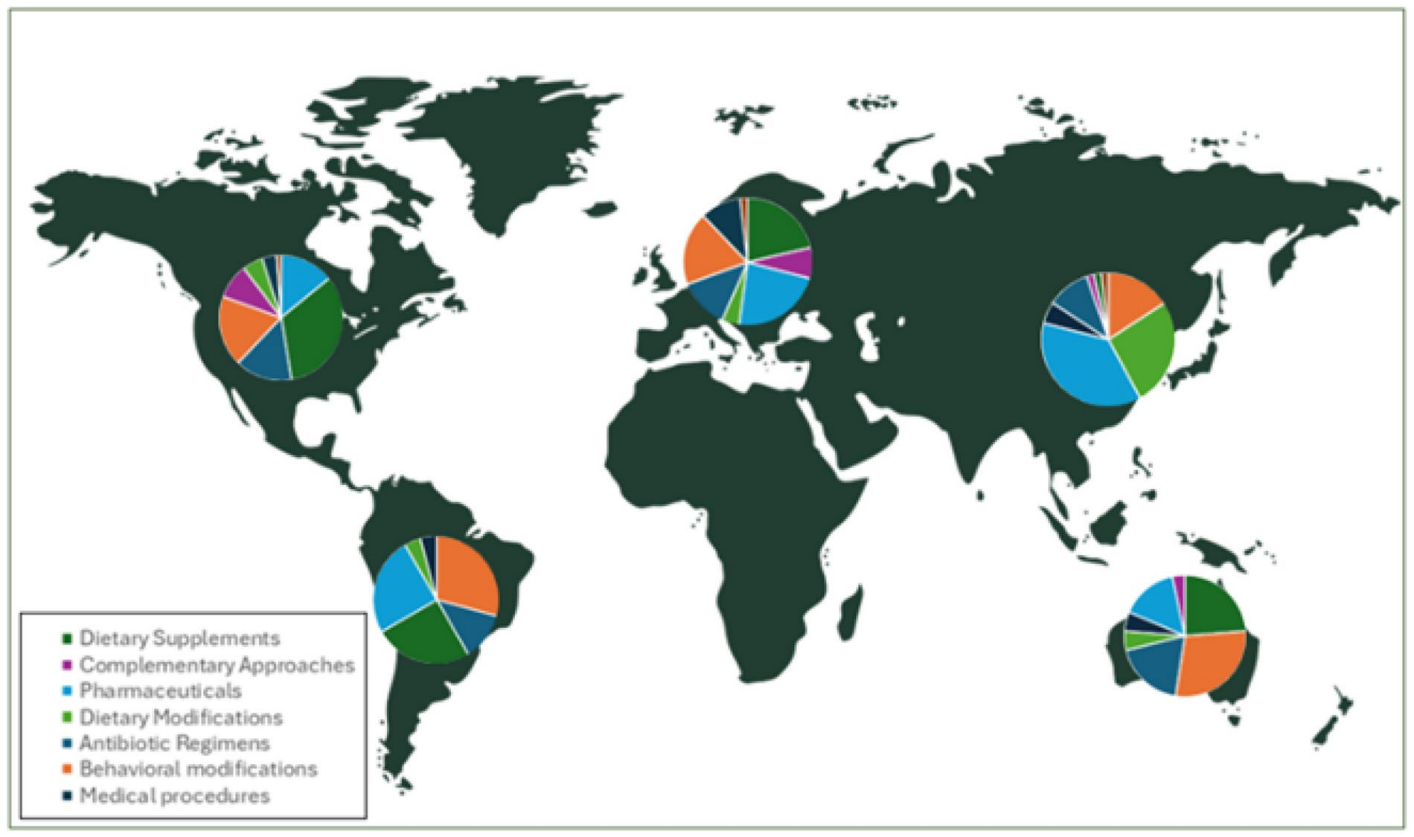
Geographical distribution of the included publications and preventive strategies for recurrent urinary tract infections in premenopausal women across continents. North America (*n* = 31), South America (*n* = 3), Europe (*n* = 33), Asia (*n* = 6), and Australia (*n* = 5).

The 78 included publications originated from 17 countries. Their geographical distribution by continent is shown in Figure 2.

### The included publications

Included publications encompassed diverse types of reviews, guidelines, and expert opinions/reviews. Through our search, we found 11 guidelines originating from Europe, North America, South America, and Australia, representing eight different countries. Furthermore, 60 diverse reviews originated from Australia, South America, Europe, and Asia, representing 14 different countries. Finally, 7 experts’ opinions/reviews originated from North America and Europe, representing four different countries. A complete overview of all included publications can be found in Table 1.

### Preventive strategies for rUTIs in premenopausal women: Distribution and expert-recommendation

We identified eight distinct categories of preventive strategies, together with multiple subcategories, for rUTIs in premenopausal women listed in Table 2.

### Pharmaceuticals

We identified four different pharmaceutical strategies.

The recommendations varied from *neutral* to *may be recommended*. The subcategory Immunostimulants (vaccines) included oral vaccines (Uro-vaxom (OM-89), vaginal vaccines (Uro-vac, Solco-urovac), injections (Strovac), intranasal vaccines, and sublingual spray vaccines (Uromune). Therefore, the average recommendation for this subcategory was an average of different kinds of vaccines, with 30 out of 69 vaccines categorised as *neutral*, 28 out of 69 as *may be recommended*, and 11 out of 69 as *recommended*. The varying recommendations are reflected in the agreement index of 13% within this preventive strategy (Table 2).

**Figure 3.**
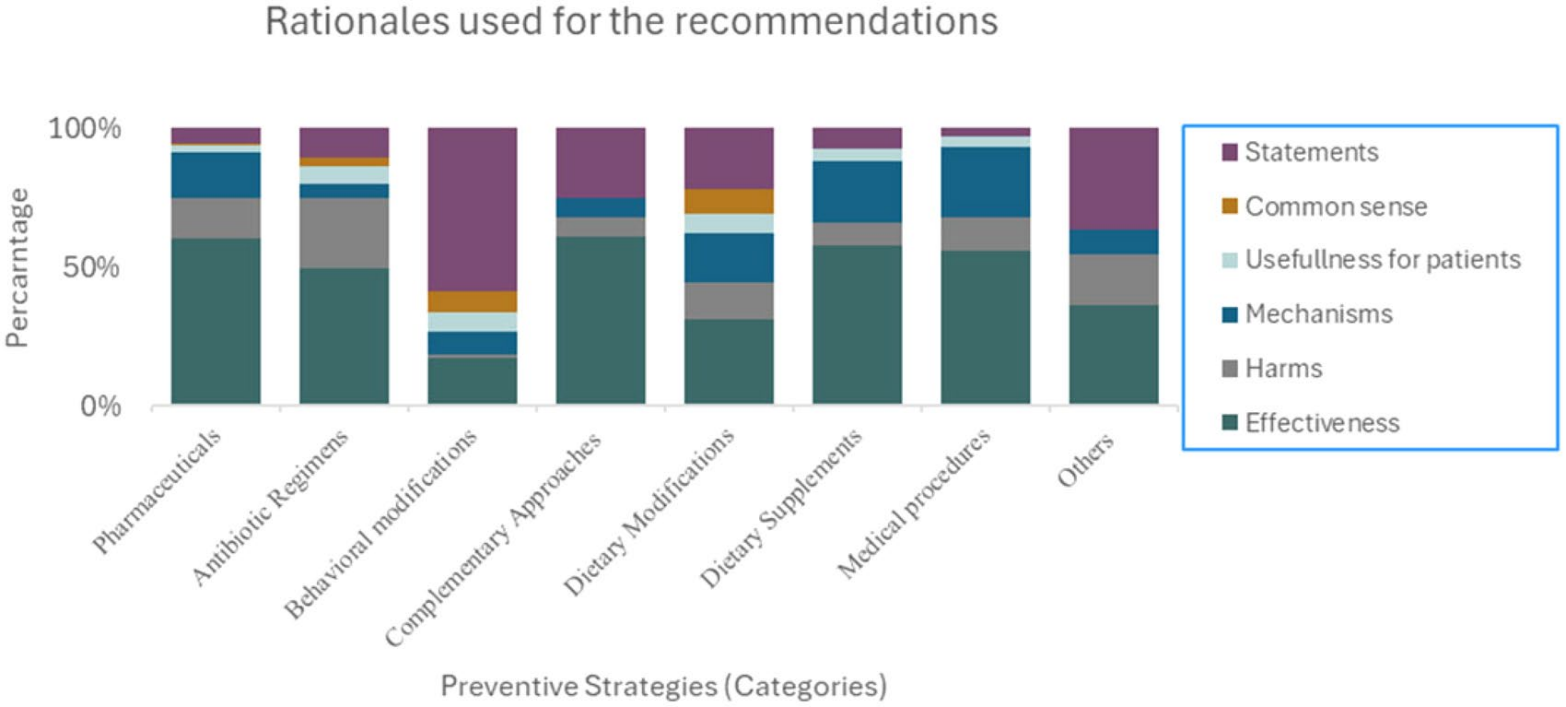
Distribution of the rationales applied across the recommendations in preventive strategies for recurrent urinary tract infections in premenopausal women. The rationales are presented as percentages within each preventive strategy category, and do not reflect the number of rationales used within each category.

The rationales for the recommendations in the pharmaceutical category mainly used arguments based on *effectiveness* (Figure 3). As for *NSAIDs*, this strategy was included in our study because it was mentioned in sections regarding preventive measures against rUTIs in the included articles, but the strategy was often recommended as an acute treatment, rather than a preventive treatment.

### Antibiotic regimens

We identified three different antibiotic regimens. The recommendations varied from *recommended* to *may be recommended* (Table 2). Agreement indices above 88% across all three antibiotic regimens point to a strong consensus concerning the recommendation within this category. The rationales for the recommendations in the antibiotic regimens category mainly used effectiveness-based arguments, but the rationale *harms* was also widely used, especially in the subcategory *Low-dose continuous antibiotic prophylaxis* (Figure 3).

### Behavioural modifications

We identified 16 different behavioural modifying strategies. The recommendations varied from *neutral to may be recommended* (Table 2). Nine out of 16 preventive strategies in this category showed agreement indices below 33%, highlighting considerable variation in expert recommendations.

The rationales for recommendations in the behavioural category predominantly relied on *statements*, while the remaining rationales were used with relatively equal frequency (Figure 3).

### Complementary approaches

We identified six different complementary strategies. The recommendation was assessed *neutral* across all subcategories, except for the strategy *Ayurveda and Unani*, which were assessed as *not recommended* (Table 2).

The rationales for the recommendations in the complementary category mainly used effectiveness-based arguments, especially articles mentioning Acupuncture and Phytotherapeutics, but the rationale *statements were* also widely used in this category (Figure 3).

### Dietary modifications

We identified two strategies concerning dietary modifications. The recommendations were assessed as *may be recommended* in both subcategories (Table 2). The recommendation for the subcategory *Specific Diet* was based on five citations, where three were categorised as *neutral*, one as *may be recommended,* and one as *recommended*. The rationales for the recommendations in the subcategory *Increased Hydration* were evenly distributed, with a slight preference for the rationale’s *effectiveness*. In contrast, in the strategy *Specific Diet*, no single rationale was more frequently employed than others. Agreement indices of 42% for the strategy *Increased hydration* and 20% for the strategy *Specific Diet* show variation in the recommendation among the authors.

### Dietary supplements

We identified nine different dietary supplementary strategies. The average recommendation varied from *may be recommended* to *neutral*. As for the subcategory *Cranberry Products,* the average recommendation relied on 20 out of 69 cranberry products categorised as *not recommended*, 26 of the 69 cranberry products as *may be recommended*, 21 out of 69 as *neutral* and only two cranberry products as *recommended.* This notable variation is reflected in the agreement index of 19% (Table 2). For D-mannose, the agreement index was 12%, highlighting significant variability in how experts recommend this preventive strategy in the literature.

The rationales for the recommendations in the dietary supplement category mainly used arguments involving statements about *effectiveness* and *mechanisms (Cranberry products*, *Probiotics*, *D-mannose,* and *Vitamins C and D).* The remaining subcategories showed a more balanced distribution of rationales, including *statements, usefulness for patients*, and *harms* (Figure 3). However, these rationales were derived from a limited number of publications (Table 2).

### Medical procedures

We identified four different medical procedures in our included articles. The recommendations varied from *may be recommended*, *neutral*, and *not recommended* (Table 2). In the subcategory *Intravesical installations*, the average recommendation was derived from 35 publications, of which 17 were categorised as *may be recommended*, 17 as *neutral*, and one as *recommended*. This distribution reflects a substantial variation in the recommendation, which is further illustrated by an agreement index of 3% (Table 2). As for *Medical procedures*, recommendations were primarily justified by statements about *effectiveness*, though the rationale *Mechanisms* were also frequently cited (Figure 3).

### Others

We identified six different strategies within the category *Others.*

The recommendations varied from *neutral* to *may be recommended* (Table 2). The rationales for the recommendations in this category were evenly distributed across *Effectiveness, Harms,* and *Statements* (Figure 3).

## Discussion

This scoping review included 78 publications, comprising systematic reviews as well as other types of reviews, expert opinion reports, and clinical guidelines that included expert recommendations on preventive strategies for rUTIs in premenopausal women. The included studies represented findings from all continents except Africa. The lack of studies from the African continent highlights broader health inequities and underscores the need for future research that supports region-specific preventive strategies, ensuring recommendations are applicable and meaningful across diverse settings.

The recommendations covered well-known and well-studied preventive strategies, such as different antibiotic regimens and cranberry products, but also covered several less-studied preventive strategies, including behavioural modifications, vitamin supplements, specific diets, and increased hydration.

Arguments for or against recommending a preventive strategy were often presented as statements without further justification. Disagreement on the recommendations was common - even when recommendations were justified by *effectiveness*.

A core finding of our scoping review was that expert recommendations for preventive strategies targeting *Behavioural modifications* were frequently based on *statements not supported by studies,* rather than *effectiveness*. This highlights the lack of evidence in the preventive area concerning behavioural modifications. Nevertheless, experts generally expressed a positive attitude towards these behavioural modifications and their potential for recommendation.

Our findings align with existing literature, which emphasises behavioural changes like personal hygiene as low-impact, first-line strategies for rUTI prevention [29–32]. However, recommendations on behavioural modifications are inconsistent. A guideline of guidelines [17] highlights variations in recommendations in guidelines regarding antibiotic-sparing preventative strategies, where some guidelines emphasise that commonly held myths surrounding lifestyle modification, such as hygiene practices, pre- and post-coital voiding, tampon use, and douching, do not play a role in rUTI prevention [30].

Consistent with existing literature, two of three specific antibiotic regimens were recommended by experts, although recommendations on postcoital antibiotics were based on rather old clinical trials [30,33–37]. *Effectiveness* and potential *Harms* were the most cited rationales, reflecting concerns in the literature about adverse events and the lack of evidence for long-term prophylactic antibiotic use [30].

Cranberries have been widely studied for UTI prevention. A Cochrane review, first published in 1998 and updated five times, most recently in 2023, now includes 50 studies [14]. While earlier versions found limited evidence, the latest supports cranberry use for rUTIs. Despite extensive research in this area, expert recommendations remain inconsistent, reflected in a low agreement index of 19% (Table 2).

Our scoping review reveals a lack of consensus on which preventive strategies should be recommended. While certain strategies, such as cranberries, have been the subject of considerable research, the evidence supporting their effectiveness has evolved. Similar shifts in recommendations may occur for other preventive strategies discussed in this scoping review as ongoing research progresses and public interest in prevention and women’s health continues to grow. However, we acknowledge that certain behavioural strategies, such as wiping patterns, cannot feasibly be evaluated through gold‑standard RCTs, which means the supporting evidence may remain less robust. This does not, in itself, invalidate the recommendations. What becomes most important moving forward is maintaining a clear awareness of these limitations.

### Strengths and limitations

This study was a scoping review following a rigorous approach in line with JBI’s guidelines for conducting scoping reviews. However, our scoping review has some limitations.

We searched five databases from January 2013 to December 2023. While limited to publications within this timespan, many included reviews likely covered studies and recommendations predating 2013.

Because the review focused specifically on preventive strategies for premenopausal women, reviews and expert papers were only included when they explicitly addressed this group. Mentions of strategies based on studies in postmenopausal women, such as oestrogen therapy, were excluded during data charting. Likewise, although guidelines could not be restricted solely to this group, strategies were excluded if the supporting studies concerned postmenopausal or pregnant women or focused on acute treatment such as for NSAID.

Because of language restrictions and the exclusion of grey literature, we may have missed region-specific recommendations. The included publications show no African representation and an overrepresentation of European and North American studies, are likely due to higher research output from these regions and our language limitations.

The framework used for the categorisation of preventive strategies was developed using qualitative content analysis. Qualitative content analysis is a validated and internationally recognised analytical method appropriate for our research question, but its use in a scoping review is novel, so we were unable to validate our approach or draw on prior studies. The majority of the included publications stemmed from biomedical research environments, which may have influenced both the focus and framing of preventive strategies, potentially favouring conventional treatments like antibiotics. This professional bias could have affected the evaluation of alternative approaches. To mitigate this, future searches should include diverse databases.

A key strength of this scoping review is that we did not limit the study to evaluating the evidence behind well-established strategies. Instead, we systematically explored experts’ perspectives, focusing on the rationale behind recommending or not recommending the strategy.

## Conclusion

This scoping review systematically identified and mapped the breadth of existing preventive strategies used for rUTIs in premenopausal women. While well-established preventive strategies were frequently recommended, a wide range of less-studied strategies were also identified. Our results demonstrate a broad and heterogeneous range of preventive strategies in this field, yet there is no expert consensus regarding the recommendation of the majority of these strategies.

To adequately help and inform this group of women, healthcare practitioners must be familiar with existing preventive strategies for rUTIs, including knowledge about where evidence is robust and where it is lacking. Our study contributes insight into which preventive strategies have been extensively researched, such as antibiotics, and where evidence is limited, as is the case for many of the behavioral strategies. This is further reflected in the fact that a substantial proportion of the rationales for these behavioral strategies were classified as *statements* rather than *effectiveness*. Furthermore, our study highlights the strategies for which there is limited expert consensus across the literature, including methenamine hippurate, D‑mannose, vaccines, increased hydration and some of the behavioral strategies. Incorporating the findings of this scoping review may help inform where future research is needed and the development of clinical guidelines, thereby facilitating their dissemination among healthcare providers.

## Declaration of generative AI and AI-assisted technologies in the manuscript preparation process

During the preparation of this work, the author used Microsoft 365′s AI-assistant Co-pilot to refine wording and assist with synonyms. After using this tool, the author reviewed and edited the content as needed and takes full responsibility for the content of the published article.

## Supplementary Material

Supplemental Material

## Figures and Tables

**Table:** 

*Effectiveness*	Encompasses studies that directly used evidence or effectiveness as the rationale for the recommendation. This category encompasses both the lack of evidence and the presence of evidence on a given preventive strategy.
***Harms***	Encompasses studies that directly used harms as the rationale behind their recommendations. ‘Harms’ includes both the absence and presence of adverse effects.
***Mechanisms***	Encompasses studies that used or mentioned the underlying mechanisms of action of the given preventive strategy.
***Usefulness***	Encompasses studies that used rationales for the recommendation based on how accessible the preventive strategy is, including, for example, costs and administration forms.
***Common sense***	This category intersects with several other categories. It encompasses rationales where studies have made recommendations based on factors such as accessibility, costs, harms, and preventive strategies that require minimal resources of the patient.
***Statements***	This category includes studies that state something about a preventive strategy without providing any rationales or arguments for the recommendation.
