# Supplementary material for: Preventive strategies for recurrent urinary tract infections in premenopausal women: A scoping review
Source: Eur J Gen Pract. 2026 May 14;32(1):2661165. doi: 10.1080/13814788.2026.2661165 (PMC13178043; doi:10.1080/13814788.2026.2661165)
Supplement: Supplemental Material [file IGEN_A_2661165_SM9419.zip › IGEN_2661165_suppl_data/ejgp-2025-0250-File005.docx]

## Appendix

#### **Search Strategy**

All included studies were exported into EndNote, where duplicates were subsequently removed. Finally, the studies were imported into Covidence.

The search string was developed by creating four conceptual groups that together represented the research question.

The search string is depicted in *Table 1,* where the four groups and their corresponding *index terms* and *MeSH*-terms are visualized. MeSH-terms were found in the MeSH database in PubMed and if additional suitable MeSH terms were found in the MeSh database in PubMed, they were added to the search string. When all four groups were created, they were merged into one single search string. A time interval from 01.01.2013-08.12.2023 was applied to the search string.

The four groups were as follows:

- Women
- Urinary tract infections
- Recurrence
- Prevention

| Table 1 | Women |  | Urinary tract infections | **Recurrence** | **Prevention** |
| --- | --- | --- | --- | --- | --- |
| **TextWords** | *Female, females, woman,*  *women* |  | ***urethritis, pyuria,***  ***pyurias,***  ***pyuria*,***  ***bacteriuria,***  ***bacteriurias,***  ***bacteriuria*,***  ***urinary tract infections,***  ***urinary tract infection*,***  ***urinary tract infection,***  ***cystitis,***  ***cystit*,***  ***bladder infection,***  ***bladder infections,***  ***bladder infection**** | ***Reinfection,***  ***reinfections,***  ***reinfection*,***  ***recurrence,***  ***recurrences,***  ***recurrence*,***  ***recurrent,***  ***recurren*,***  ***recurring,***  ***reoccurrence,***  ***relapse,***  ***relapses,***  ***relaps*,***  ***relapsing,***  ***reappearance,***  ***reappearances,***  ***reappearance*,***  ***chronic,***  ***chronically,***  ***chronical,***  ***chronic**** | ***disease management,***  ***secondary prevention,***  ***prevention,*** ***preventive,***  ***preventative, preventions,*** ***preventi*,***  ***preventive measures,***  ***preventive measure,***  ***preventive strategies,***  ***preventive strategy,***  ***preventive strateg*,***  ***preventive therapy,***  ***preventive therapies,***  ***preventive therap*,***  ***preventive measure*,***  ***profylaxis,*** ***prophylax,***  ***prophylaxes, prophylaxy,*** ***prophylactic,***  ***prophyla*, ”prevention and control”*** |
| **MeSH-terms** | *female, women* |  | ***urethritis, pyuria, bacteriuria, urinary tract infections, cystitis*** | ***Reinfection,***  ***recurrence*** | ***disease management,***  ***secondary prevention*** |
| **Subheading** |  |  |  |  | ***Prevention and control*** |
