# Supplementary material for: Preventive strategies for recurrent urinary tract infections in premenopausal women: A scoping review
Source: Eur J Gen Pract. 2026 May 14;32(1):2661165. doi: 10.1080/13814788.2026.2661165 (PMC13178043; doi:10.1080/13814788.2026.2661165)
Supplement: Supplemental Material [file IGEN_A_2661165_SM9419.zip › IGEN_2661165_suppl_data/ejgp-2025-0250-File006.docx]

| **First author** | **Overgroup** | **Subgroup** |
| --- | --- | --- |
| Abou Heidar, N. | Behavioral modifications | Limiting use of spermicides |
|  | Behavioral modifications | Behavioral modifications (non specified) |
|  | Dietary Supplements | Cranberry products |
|  | Pharmaceuticals | Antibiotics |
|  | Pharmaceuticals | Antibiotics |
|  | Medical procedures | Intravesical installations |
|  | Dietary Supplements | Probiotics (different Lactobacilli) |
|  | Pharmaceuticals | Antibiotics |
|  | Pharmaceuticals | Immunostimulants (vaccines) |
|  | Behavioral modifications | Proper wiping patterns |
|  | Pharmaceuticals | Antibiotics |
|  | Pharmaceuticals | Antibiotics |
|  | Dietary Supplements | Other alkalizing agents |
|  | Antibiotic Regimens | Low dose continuous antibiotic prophylaxis |
| Al-Badr, A. | Complementary Approaches | Acupuncture |
|  | Antibiotic Regimens | Self-start antibiotics |
|  | Pharmaceuticals | Antibiotics |
|  | Behavioral modifications | Behavioral modifications (non specified) |
|  | Behavioral modifications | Avoiding skin allergens (soaps, vaginals creams, bubble baths, hot tubs etc.) |
|  | Behavioral modifications | Avoiding intercourse with multiple sexual partners |
|  | Antibiotic Regimens | Low dose continuous antibiotic prophylaxis |
|  | Dietary Supplements | Cranberry products |
|  | Dietary Modifications | Increased hydration |
|  | Dietary Supplements | Probiotics (different Lactobacilli) |
|  | Pharmaceuticals | Anti-infective: Methenamine Hippurate |
|  | Pharmaceuticals | Immunostimulants (vaccines) |
|  | Antibiotic Regimens | Postcoital antibiotic prophylaxis |
|  | Behavioral modifications | Cleaning genital areas before and after sexual intercourse |
| Anger, J. | Pharmaceuticals | Antibiotics |
|  | Dietary Supplements | Cranberry products |
| Anger, J. T. | Antibiotic Regimens | Low dose continuous antibiotic prophylaxis |
|  | Dietary Supplements | Cranberry products |
|  | Pharmaceuticals | Anti-infective: Methenamine Hippurate |
| Aragón, I. M. | Dietary Supplements | Cranberry products |
|  | Dietary Supplements | D-mannose |
|  | Dietary Supplements | Probiotics (different Lactobacilli) |
| Arnold, James J. | Antibiotic Regimens | Postcoital antibiotic prophylaxis |
|  | Antibiotic Regimens | Low dose continuous antibiotic prophylaxis |
|  | Dietary Supplements | Cranberry products |
| Aslam, S. | Behavioral modifications | Avoiding postponing urination |
|  | Dietary Supplements | L-arginine |
|  | Antibiotic Regimens | Postcoital antibiotic prophylaxis |
|  | Dietary Supplements | Cranberry products |
|  | Dietary Supplements | D-mannose |
|  | Complementary Approaches | Phytoterapeutics |
|  | Dietary Modifications | Increased hydration |
|  | Dietary Supplements | Probiotics (different Lactobacilli) |
|  | Behavioral modifications | Behavioral modifications (non specified) |
|  | Dietary Supplements | Probiotics (different Lactobacilli) |
|  | Dietary Supplements | Vitamins (C and D) |
| Barber, A. E. | Pharmaceuticals | Antibiotics |
|  | Dietary Supplements | D-mannose |
|  | Dietary Supplements | Anti-adhesive therapeutics: Pillicides and mannosides |
|  | Pharmaceuticals | Immunostimulants (vaccines) |
| Barclay, J. | Dietary Supplements | Cranberry products |
|  | Dietary Supplements | D-mannose |
|  | Antibiotic Regimens | Low dose continuous antibiotic prophylaxis |
|  | Pharmaceuticals | Anti-infective: Methenamine Hippurate |
|  | Pharmaceuticals | Immunostimulants (vaccines) |
|  | Dietary Supplements | Probiotics (different Lactobacilli) |
|  | Dietary Supplements | Other alkalizing agents |
| Barea, B. M. | Complementary Approaches | Acupuncture |
|  | Complementary Approaches | Chinese herbal medicines |
|  | Others | Combination of products |
|  | Dietary Supplements | Cranberry products |
|  | Dietary Supplements | D-mannose |
|  | Medical procedures | Intravesical installations |
|  | Pharmaceuticals | Immunostimulants (vaccines) |
|  | Pharmaceuticals | Anti-infective: Methenamine Hippurate |
|  | Pharmaceuticals | NSAID |
|  | Dietary Supplements | Probiotics (different Lactobacilli) |
|  | Dietary Supplements | Other alkalizing agents |
|  | Dietary Supplements | Vitamins (C and D) |
| Beerepoot, M. A. | Complementary Approaches | Acupuncture |
|  | Dietary Supplements | Cranberry products |
|  | Dietary Supplements | D-mannose |
|  | Pharmaceuticals | Immunostimulants (vaccines) |
|  | Pharmaceuticals | Immunostimulants (vaccines) |
|  | Dietary Supplements | Vitamins (C and D) |
| Beerepoot, M. | Complementary Approaches | Phytoterapeutics |
|  | Complementary Approaches | Acupuncture |
|  | Dietary Supplements | Cranberry products |
|  | Dietary Supplements | Probiotics (different Lactobacilli) |
|  | Dietary Supplements | Probiotics (different Lactobacilli) |
|  | Pharmaceuticals | Immunostimulants (vaccines) |
|  | Pharmaceuticals | Immunostimulants (vaccines) |
| Bergamin, P. A. | Dietary Supplements | Vitamins (C and D) |
|  | Behavioral modifications | Behavioral modifications (non specified) |
|  | Behavioral modifications | Avoiding obesity |
|  | Antibiotic Regimens | Low dose continuous antibiotic prophylaxis |
|  | Dietary Supplements | Cranberry products |
|  | Dietary Supplements | D-mannose |
|  | Dietary Modifications | Increased hydration |
|  | Medical procedures | Intravesical installations |
|  | Medical procedures | Intravesical installations |
|  | Pharmaceuticals | Anti-infective: Methenamine Hippurate |
|  | Antibiotic Regimens | Postcoital antibiotic prophylaxis |
|  | Behavioral modifications | Complete bladder emptying |
|  | Dietary Supplements | Probiotics (different Lactobacilli) |
|  | Antibiotic Regimens | Self-start antibiotics |
|  | Behavioral modifications | Proper wiping patterns |
|  | Pharmaceuticals | Immunostimulants (vaccines) |
| Betschart, C. | Pharmaceuticals | Anti-infective: Methenamine Hippurate |
|  | Dietary Modifications | Increased hydration |
|  | Complementary Approaches | Phytoterapeutics |
|  | Antibiotic Regimens | Low dose continuous antibiotic prophylaxis |
|  | Behavioral modifications | Limiting use of spermicides |
|  | Behavioral modifications | Avoiding hypothermia |
|  | Behavioral modifications | Proper wiping patterns |
|  | Dietary Supplements | Cranberry products |
|  | Dietary Supplements | D-mannose |
|  | Medical procedures | Intravesical installations |
|  | Behavioral modifications | Avoiding skin allergens (soaps, vaginals creams, bubble baths, hot tubs etc.) |
|  | Pharmaceuticals | Immunostimulants (vaccines) |
|  | Complementary Approaches | Phytoterapeutics |
| Brubaker, L. | Medical procedures | Intravesical installations |
|  | Dietary Supplements | Vitamins (C and D) |
|  | Complementary Approaches | Chinese herbal medicines |
|  | Pharmaceuticals | Antibiotics |
|  | Antibiotic Regimens | Low dose continuous antibiotic prophylaxis |
|  | Dietary Supplements | Cranberry products |
|  | Dietary Supplements | D-mannose |
|  | Pharmaceuticals | Immunostimulants (vaccines) |
|  | Pharmaceuticals | Anti-infective: Methenamine Hippurate |
|  | Medical procedures | Intravesical installations |
|  | Antibiotic Regimens | Postcoital antibiotic prophylaxis |
|  | Dietary Supplements | Probiotics (different Lactobacilli) |
| Cai, T | Complementary Approaches | Chinese herbal medicines |
|  | Dietary Supplements | Cranberry products |
|  | Dietary Supplements | D-mannose |
|  | Dietary Supplements | Probiotics (different Lactobacilli) |
| Caron, F. | Dietary Modifications | Increased hydration |
|  | Behavioral modifications | Avoiding postponing urination |
|  | Others | Improving bowel movement |
|  | Behavioral modifications | Limiting use of spermicides |
|  | Dietary Supplements | Cranberry Products |
|  | Antibiotic Regimens | Low dose continuous antibiotic prophylaxis |
|  | Antibiotic Regimens | Postcoital antibiotic prophylaxis |
| Chen, Y. C. | Dietary Supplements | Cranberry products |
|  | Dietary Supplements | D-mannose |
|  | Medical procedures | Intravesical installations |
|  | Pharmaceuticals | Anti-infective: Methenamine Hippurate |
|  | Dietary Supplements | Probiotics (different Lactobacilli) |
|  | Pharmaceuticals | Immunostimulants (vaccines) |
| Chetwood, A. | Behavioral modifications | Limiting use of spermicides |
|  | Dietary Supplements | Cranberry products |
|  | Medical procedures | Intravesical installations |
|  | Behavioral modifications | Behavioral modifications (non specified) |
|  | Antibiotic Regimens | Low dose continuous antibiotic prophylaxis |
|  | Dietary Modifications | Specific diet |
|  | Antibiotic Regimens | Postcoital antibiotic prophylaxis |
|  | Antibiotic Regimens | Self-start antibiotics |
|  | Medical procedures | Urethral dilation |
|  | Pharmaceuticals | Immunostimulants (vaccines) |
| Christofides, A. | Behavioral modifications | Behavioral modifications (non specified) |
|  | Dietary Supplements | Cranberry products |
|  | Medical procedures | Intravesical installations |
|  | Antibiotic Regimens | Low dose continuous antibiotic prophylaxis |
|  | Pharmaceuticals | Anti-infective: Methenamine Hippurate |
|  | Complementary Approaches | Phytoterapeutics |
|  | Pharmaceuticals | Immunostimulants (vaccines) |
|  | Dietary Supplements | Probiotics (different Lactobacilli) |
|  | Antibiotic Regimens | Self-start antibiotics |
|  | Antibiotic Regimens | Postcoital antibiotic prophylaxis |
|  | Medical procedures | Urethral dilation |
|  | Dietary Supplements | Probiotics (different Lactobacilli) |
| Costantini, E. | Complementary Approaches | Acupuncture |
|  | Complementary Approaches | Chinese herbal medicines |
|  | Others | Combination of products |
|  | Dietary Supplements | Cranberry products |
|  | Dietary Supplements | D-mannose |
|  | Medical procedures | Intravesical installations |
|  | Pharmaceuticals | Anti-infective: Methenamine Hippurate |
|  | Pharmaceuticals | Immunostimulants (vaccines) |
|  | Pharmaceuticals | Immunostimulants (vaccines) |
|  | Dietary Supplements | Probiotics (different Lactobacilli) |
|  | Pharmaceuticals | Immunostimulants (vaccines) |
| Da Silva, A. L. | Behavioral modifications | Behavioral modifications (non specified) |
|  | Antibiotic Regimens | Low dose continuous antibiotic prophylaxis |
|  | Dietary Supplements | Cranberry products |
|  | Dietary Supplements | Probiotics (different Lactobacilli) |
|  | Pharmaceuticals | Immunostimulants (vaccines) |
|  | Antibiotic Regimens | Self-start antibiotics |
|  | Pharmaceuticals | Immunostimulants (vaccines) |
| De Rossi, P. | Behavioral modifications | Avoiding postponing urination |
|  | Behavioral modifications | Avoiding skin allergens (soaps, vaginals creams, bubble baths, hot tubs etc.) |
|  | Dietary Supplements | Cranberry products |
|  | Behavioral modifications | Wear cotton underwear |
|  | Pharmaceuticals | Immunostimulants (vaccines) |
|  | Dietary Modifications | Increased hydration |
|  | Behavioral modifications | Postcoital-voiding |
|  | Antibiotic Regimens | Postcoital antibiotic prophylaxis |
|  | Behavioral modifications | Proper wiping patterns |
| Epp, A. | Complementary Approaches | Acupuncture |
|  | Antibiotic Regimens | Low dose continuous antibiotic prophylaxis |
|  | Dietary Supplements | Cranberry products |
|  | Behavioral modifications | Behavioral modifications (non specified) |
|  | Antibiotic Regimens | Postcoital antibiotic prophylaxis |
|  | Dietary Supplements | Probiotics (different Lactobacilli) |
|  | Antibiotic Regimens | Self-start antibiotics |
|  | Others | Topical hyaluronic acid |
|  | Pharmaceuticals | Immunostimulants (vaccines) |
| Farford, B.58 | Complementary Approaches | Acupuncture |
|  | Behavioral modifications | Behavioral modifications (non specified) |
|  | Antibiotic Regimens | Low dose continuous antibiotic prophylaxis |
|  | Dietary Supplements | Cranberry products |
|  | Dietary Supplements | D-mannose |
|  | Pharmaceuticals | Anti-infective: Methenamine Hippurate |
|  | Pharmaceuticals | Immunostimulants (vaccines) |
|  | Antibiotic Regimens | Postcoital antibiotic prophylaxis |
|  | Dietary Supplements | Probiotics (different Lactobacilli) |
|  | Antibiotic Regimens | Self-start antibiotics |
|  | Pharmaceuticals | Immunostimulants (vaccines) |
| Feng, Feng | Complementary Approaches | Acupuncture |
|  | Complementary Approaches | Chinese herbal medicines |
|  | Dietary Supplements | Cranberry products |
|  | Dietary Modifications | Specific diet |
|  | Dietary Supplements | D-mannose |
|  | Dietary Modifications | Increased hydration |
|  | Complementary Approaches | Massage and Touch therapies, reflexology |
|  | Complementary Approaches | Ayurveda and Unani |
|  | Complementary Approaches | Massage and Touch therapies, reflexology |
|  | Complementary Approaches | Phytoterapeutics |
|  | Dietary Supplements | Vitamins (C and D) |
|  | Complementary Approaches | Yoga |
| Finney, E. L. | Complementary Approaches | Acupuncture |
|  | Dietary Supplements | Cranberry products |
|  | Dietary Modifications | Specific diet |
|  | Dietary Supplements | D-mannose |
|  | Dietary Modifications | Increased hydration |
|  | Complementary Approaches | Phytoterapeutics |
|  | Medical procedures | Intravesical installations |
|  | Behavioral modifications | Proper wiping patterns |
|  | Dietary Supplements | Probiotics (different Lactobacilli) |
|  | Dietary Supplements | Vitamins (C and D) |
|  | Dietary Supplements | Vitamins (C and D) |
| Foxman, B. | Complementary Approaches | Phytoterapeutics |
|  | Others | Bacteriophages |
|  | Dietary Supplements | Cranberry Products |
|  | Complementary Approaches | Phytoterapeutics |
|  | Complementary Approaches | Phytoterapeutics |
|  | Dietary Supplements | Probiotics (different Lactobacilli) |
|  | Complementary Approaches | Phytoterapeutics |
|  | Pharmaceuticals | Immunostimulants (vaccines) |
| Geerlings, S. E. | Pharmaceuticals | Antibiotics |
|  | Dietary Supplements | Cranberry products |
|  | Dietary Supplements | Probiotics (different Lactobacilli) |
|  | Pharmaceuticals | Anti-infective: Methenamine Hippurate |
|  | Pharmaceuticals | Immunostimulants (vaccines) |
|  | Antibiotic Regimens | Self-start antibiotics |
|  | Pharmaceuticals | Immunostimulants (vaccines) |
|  | Dietary Supplements | Vitamins (C and D) |
| Glover, E. K. | Pharmaceuticals | Antibiotics |
|  | Pharmaceuticals | Anti-infective: Methenamine Hippurate |
| Glover, M. | Pharmaceuticals | Antibiotics |
|  | Dietary Supplements | Cranberry products |
|  | Antibiotic Regimens | Postcoital antibiotic prophylaxis |
| Guglietta, A. | Dietary Supplements | Cranberry products |
|  | Others | Intestinal mucosal barrier agents |
|  | Dietary Supplements | Probiotics (different Lactobacilli) |
|  | Antibiotic Regimens | Low dose continuous antibiotic prophylaxis |
| Gupta, Kalpana | Pharmaceuticals | Antibiotics |
|  | Dietary Supplements | Cranberry products |
|  | Dietary Supplements | Probiotics (different Lactobacilli) |
| Haddad, J. M. | Dietary Supplements | Cranberry products |
|  | Dietary Supplements | D-mannose |
|  | Behavioral modifications | Behavioral modifications (non specified) |
|  | Medical procedures | Intravesical installations |
|  | Pharmaceuticals | Immunostimulants (vaccines) |
|  | Pharmaceuticals | Anti-infective: Methenamine Hippurate |
|  | Dietary Supplements | Probiotics (different Lactobacilli) |
|  | Pharmaceuticals | Antibiotics |
| Harding, C. | Pharmaceuticals | Antibiotics |
|  | Dietary Supplements | D-mannose |
|  | Medical procedures | Intravesical installations |
|  | Pharmaceuticals | Immunostimulants (vaccines) |
| Hernandez-Hernandez, D. | Pharmaceuticals | Antibiotics |
|  | Pharmaceuticals | Immunostimulants (vaccines) |
| Hickling, D. R. | Antibiotic Regimens | Self-start antibiotics |
|  | Dietary Supplements | Vitamins (C and D) |
|  | Behavioral modifications | Behavioral modifications (non specified) |
|  | Antibiotic Regimens | Low dose continuous antibiotic prophylaxis |
|  | Dietary Supplements | Cranberry products |
|  | Dietary Supplements | D-mannose |
|  | Medical procedures | Intravesical installations |
|  | Pharmaceuticals | Anti-infective: Methenamine Hippurate |
|  | Antibiotic Regimens | Postcoital antibiotic prophylaxis |
|  | Dietary Supplements | Probiotics (different Lactobacilli) |
|  | Pharmaceuticals | Immunostimulants (vaccines) |
| Hutton, H.; Amos, L. | Pharmaceuticals | Anti-infective: Methenamine Hippurate |
|  | Behavioral modifications | Behavioral modifications (non specified) |
|  | Antibiotic Regimens | Low dose continuous antibiotic prophylaxis |
|  | Dietary Supplements | Cranberry products |
|  | Antibiotic Regimens | Self-start antibiotics |
|  | Antibiotic Regimens | Postcoital antibiotic prophylaxis |
|  | Dietary Supplements | Probiotics (different Lactobacilli) |
|  | Dietary Supplements | Other alkalizing agents |
| Jarvis, T. R. | Dietary Supplements | Other alkalizing agents |
|  | Behavioral modifications | Complete bladder emptying |
|  | Dietary Supplements | Cranberry products |
|  | Antibiotic Regimens | Low dose continuous antibiotic prophylaxis |
|  | Antibiotic Regimens | Self-start antibiotics |
| Jhang, J. F. | Pharmaceuticals | Antibiotics |
|  | Dietary Supplements | Cranberry products |
|  | Pharmaceuticals | Immunostimulants (vaccines) |
|  | Dietary Supplements | Probiotics (different Lactobacilli) |
| Kolman, K. B. | Antibiotic Regimens | Low dose continuous antibiotic prophylaxis |
|  | Dietary Supplements | Cranberry products |
|  | Dietary Supplements | D-mannose |
|  | Medical procedures | Intravesical installations |
|  | Dietary Supplements | Probiotics (different Lactobacilli) |
| Kranz, J. | Behavioral modifications | Behavioral modifications (non specified) |
|  | Antibiotic Regimens | Low dose continuous antibiotic prophylaxis |
|  | Pharmaceuticals | Immunostimulants (vaccines) |
|  | Dietary Supplements | D-mannose |
|  | Pharmaceuticals | Immunostimulants (vaccines) |
|  | Complementary Approaches | Phytoterapeutics |
|  | Antibiotic Regimens | Postcoital antibiotic prophylaxis |
| Kranz, J. | Complementary Approaches | Acupuncture |
|  | Behavioral modifications | Behavioral modifications (non specified) |
|  | Dietary Supplements | Cranberry products |
|  | Dietary Supplements | D-mannose |
|  | Pharmaceuticals | Immunostimulants (vaccines) |
|  | Medical procedures | Intravesical installations |
|  | Dietary Supplements | Probiotics (different Lactobacilli) |
|  | Antibiotic Regimens | Low dose continuous antibiotic prophylaxis |
|  | Pharmaceuticals | Immunostimulants (vaccines) |
|  | Complementary Approaches | Phytoterapeutics |
|  | Antibiotic Regimens | Postcoital antibiotic prophylaxis |
|  | Pharmaceuticals | Immunostimulants (vaccines) |
| Krishnaswamy, P. H. | Pharmaceuticals | Antibiotics |
|  | Pharmaceuticals | Antibiotics |
|  | Behavioral modifications | Complete bladder emptying |
|  | Behavioral modifications | Wear cotton underwear |
|  | Dietary Supplements | Cranberry products |
|  | Dietary Supplements | D-mannose |
|  | Behavioral modifications | Behavioral modifications (non specified) |
|  | Medical procedures | Intravesical installations |
|  | Pharmaceuticals | Immunostimulants (vaccines) |
|  | Pharmaceuticals | Anti-infective: Methenamine Hippurate |
|  | Dietary Supplements | Probiotics (different Lactobacilli) |
| Kucheria, A. | Dietary Modifications | Increased hydration |
|  | Behavioral modifications | Postcoital-voiding |
|  | Behavioral modifications | Behavioral modifications (non specified) |
|  | Dietary Supplements | Cranberry products |
|  | Dietary Supplements | D-mannose |
|  | Pharmaceuticals | Anti-infective: Methenamine Hippurate |
|  | Medical procedures | Intravesical installations |
|  | Pharmaceuticals | Immunostimulants (vaccines) |
|  | Dietary Supplements | Probiotics (different Lactobacilli) |
|  | Pharmaceuticals | Immunostimulants (vaccines) |
| Kwok, M. | Complementary Approaches | Acupuncture |
|  | Behavioral modifications | Limiting use of spermicides |
|  | Behavioral modifications | Behavioral modifications (non specified) |
|  | Antibiotic Regimens | Low dose continuous antibiotic prophylaxis |
|  | Dietary Supplements | Cranberry products |
|  | Dietary Supplements | D-mannose |
|  | Pharmaceuticals | Immunostimulants (vaccines) |
|  | Dietary Modifications | Increased hydration |
|  | Medical procedures | Intravesical installations |
|  | Pharmaceuticals | Anti-infective: Methenamine Hippurate |
|  | Complementary Approaches | Phytoterapeutics |
|  | Antibiotic Regimens | Postcoital antibiotic prophylaxis |
|  | Dietary Supplements | Probiotics (different Lactobacilli) |
|  | Antibiotic Regimens | Self-start antibiotics |
|  | Pharmaceuticals | NSAID |
| Lee, D. S. | Pharmaceuticals | Immunostimulants (vaccines) |
|  | Behavioral modifications | Complete bladder emptying |
| Liska, D. | Dietary Supplements | Cranberry products |
|  | Dietary Supplements | Probiotics (different Lactobacilli) |
| Lodhia, S. | Behavioral modifications | Avoiding obesity |
|  | Behavioral modifications | Avoiding sexual intercourse |
|  | Behavioral modifications | Avoiding skin allergens (soaps, vaginals creams, bubble baths, hot tubs etc.) |
|  | Behavioral modifications | Avoiding skin allergens (soaps, vaginals creams, bubble baths, hot tubs etc.) |
|  | Behavioral modifications | Limiting use of spermicides |
|  | Dietary Supplements | Cranberry products |
|  | Dietary Supplements | D-mannose |
|  | Pharmaceuticals | Immunostimulants (vaccines) |
|  | Medical procedures | Intravesical installations |
|  | Antibiotic Regimens | Low dose continuous antibiotic prophylaxis |
|  | Pharmaceuticals | Anti-infective: Methenamine Hippurate |
|  | Antibiotic Regimens | Postcoital antibiotic prophylaxis |
|  | Dietary Supplements | Probiotics (different Lactobacilli) |
|  | Antibiotic Regimens | Self-start antibiotics |
|  | Medical procedures | Urethral dilation |
|  | Behavioral modifications | Postcoital-voiding |
|  | Behavioral modifications | Wear cotton underwear |
|  | Behavioral modifications | Proper wiping patterns |
| Martell, J. A. O. | Antibiotic Regimens | Low dose continuous antibiotic prophylaxis |
|  | Complementary Approaches | Acupuncture |
|  | Behavioral modifications | Behavioral modifications (non specified) |
|  | Dietary Supplements | Cranberry products |
|  | Dietary Supplements | D-mannose |
|  | Pharmaceuticals | Immunostimulants (vaccines) |
|  | Antibiotic Regimens | Postcoital antibiotic prophylaxis |
|  | Dietary Supplements | Probiotics (different Lactobacilli) |
|  | Behavioral modifications | Limiting use of spermicides |
| Martin, Christy D. | Dietary Supplements | Cranberry products |
|  | Dietary Supplements | D-mannose |
|  | Pharmaceuticals | Immunostimulants (vaccines) |
| Miranne, J. M. | Others | Addressing concomitant fecal incontinence |
|  | Antibiotic Regimens | Low dose continuous antibiotic prophylaxis |
|  | Antibiotic Regimens | Postcoital antibiotic prophylaxis |
|  | Behavioral modifications | Limiting use of spermicides |
|  | Dietary Modifications | Specific diet |
|  | Behavioral modifications | Proper wiping patterns |
|  | Dietary Supplements | Cranberry products |
|  | Dietary Supplements | D-mannose |
|  | Dietary Modifications | Increased hydration |
|  | Behavioral modifications | Avoiding tampons/prolonged use of pads |
|  | Pharmaceuticals | Anti-infective: Methenamine Hippurate |
|  | Behavioral modifications | Avoiding spermicide-based contraceptives (diaphragm use, condoms etc.) |
|  | Dietary Supplements | Probiotics (different Lactobacilli) |
|  | Behavioral modifications | Avoiding skin allergens (soaps, vaginals creams, bubble baths, hot tubs etc.) |
|  | Behavioral modifications | Avoiding postponing urination |
|  | Dietary Supplements | Vitamins (C and D) |
|  | Behavioral modifications | Postcoital-voiding |
|  | Behavioral modifications | Proper wiping patterns |
| Moussa, M. | Antibiotic Regimens | Low dose continuous antibiotic prophylaxis |
|  | Others | Bacteriophages |
|  | Behavioral modifications | Behavioral modifications (non specified) |
|  | Dietary Supplements | Cranberry products |
|  | Dietary Supplements | D-mannose |
|  | Pharmaceuticals | Antibiotics |
|  | Medical procedures | Intravesical installations |
|  | Pharmaceuticals | Antibiotics |
|  | Pharmaceuticals | Immunostimulants (vaccines) |
|  | Pharmaceuticals | Immunostimulants (vaccines) |
|  | Pharmaceuticals | Immunostimulants (vaccines) |
|  | Antibiotic Regimens | Postcoital antibiotic prophylaxis |
|  | Dietary Supplements | Probiotics (different Lactobacilli) |
|  | Pharmaceuticals | Antibiotics |
|  | Pharmaceuticals | Anti-infective: Methenamine Hippurate |
|  | Dietary Supplements | Vitamins (C and D) |
|  | Dietary Supplements | Vitamins (C and D) |
| Negus, M. | Complementary Approaches | Chinese herbal medicines |
|  | Dietary Supplements | Cranberry products |
|  | Dietary Supplements | D-mannose |
|  | Medical procedures | Intravesical installations |
|  | Dietary Supplements | Probiotics (different Lactobacilli) |
|  | Antibiotic Regimens | Low dose continuous antibiotic prophylaxis |
|  | Pharmaceuticals | Anti-infective: Methenamine Hippurate |
|  | Medical procedures | Urethral dilation |
|  | Pharmaceuticals | Immunostimulants (vaccines) |
| Nikpoor, P. | Pharmaceuticals | Antibiotics |
|  | Behavioral modifications | Avoiding intercourse with multiple sexual partners |
|  | Behavioral modifications | Limiting use of spermicides |
|  | Behavioral modifications | Avoiding spermicide-based contraceptives (diaphragm use, condoms etc.) |
|  | Behavioral modifications | Avoiding skin allergens (soaps, vaginals creams, bubble baths, hot tubs etc.) |
|  | Behavioral modifications | Complete bladder emptying |
|  | Dietary Supplements | Cranberry products |
|  | Pharmaceuticals | Immunostimulants (vaccines) |
|  | Dietary Modifications | Increased hydration |
|  | Behavioral modifications | Proper wiping patterns |
|  | Pharmaceuticals | Anti-infective: Methenamine Hippurate |
|  | Behavioral modifications | Proper wiping patterns |
|  | Dietary Supplements | Probiotics (different Lactobacilli) |
|  | Behavioral modifications | Avoiding postponing urination |
|  | Behavioral modifications | Wear cotton underwear |
|  | Behavioral modifications | Proper wiping patterns |
| O'Brien, V. P. | Dietary Supplements | Cranberry products |
|  | Dietary Supplements | Glycolipids/galactocides |
|  | Medical procedures | Intravesical installations |
|  | Dietary Supplements | D-mannose |
|  | Dietary Supplements | Anti-adhesive therapeutics: Pillicides and mannosides |
|  | Dietary Supplements | Probiotics (different Lactobacilli) |
|  | Pharmaceuticals | Immunostimulants (vaccines) |
|  | Pharmaceuticals | Immunostimulants (vaccines) |
| O'Riordan | Behavioral modifications | Avoiding spermicide-based contraceptives (diaphragm use, condoms etc.) |
|  | Behavioral modifications | Avoiding sexual intercourse |
|  | Behavioral modifications | Limiting use of spermicides |
|  | Dietary Supplements | Cranberry products |
|  | Dietary Supplements | D-mannose |
|  | Pharmaceuticals | Immunostimulants (vaccines) |
|  | Dietary Modifications | Increased hydration |
|  | Medical procedures | Intravesical installations |
|  | Medical procedures | Intravesical installations |
|  | Antibiotic Regimens | Low dose continuous antibiotic prophylaxis |
|  | Pharmaceuticals | Anti-infective: Methenamine Hippurate |
|  | Antibiotic Regimens | Postcoital antibiotic prophylaxis |
|  | Behavioral modifications | Postcoital-voiding |
|  | Antibiotic Regimens | Self-start antibiotics |
|  | Behavioral modifications | Avoiding postponing urination |
|  | Medical procedures | Urethral dilation |
|  | Behavioral modifications | Proper wiping patterns |
|  | Behavioral modifications | Wear cotton underwear |
| Panesar, K. | Behavioral modifications | Avoiding skin allergens (soaps, vaginals creams, bubble baths, hot tubs etc.) |
|  | Behavioral modifications | Limiting use of spermicides |
|  | Behavioral modifications | Wear cotton underwear |
|  | Dietary Supplements | Cranberry products |
|  | Dietary Supplements | D-mannose |
|  | Behavioral modifications | Avoiding postponing urination |
|  | Dietary Modifications | Increased hydration |
|  | Pharmaceuticals | Anti-infective: Methenamine Hippurate |
|  | Antibiotic Regimens | Postcoital antibiotic prophylaxis |
|  | Behavioral modifications | Avoiding sexual intercourse |
|  | Antibiotic Regimens | Self-start antibiotics |
|  | Behavioral modifications | Postcoital-voiding |
|  | Pharmaceuticals | Immunostimulants (vaccines) |
|  | Behavioral modifications | Proper wiping patterns |
| Peck, J. | Complementary Approaches | Acupuncture |
|  | Behavioral modifications | Limiting use of spermicides |
|  | Complementary Approaches | Chinese herbal medicines |
|  | Dietary Supplements | Cranberry products |
|  | Antibiotic Regimens | Low dose continuous antibiotic prophylaxis |
|  | Dietary Supplements | D-mannose |
|  | Dietary Modifications | Increased hydration |
|  | Behavioral modifications | Avoiding postponing urination |
|  | Medical procedures | Intravesical installations |
|  | Dietary Supplements | Probiotics (different Lactobacilli) |
|  | Pharmaceuticals | Anti-infective: Methenamine Hippurate |
|  | Antibiotic Regimens | Postcoital antibiotic prophylaxis |
|  | Behavioral modifications | Avoiding tampons/prolonged use of pads |
|  | Pharmaceuticals | Immunostimulants (vaccines) |
|  | Behavioral modifications | Proper wiping patterns |
|  | Dietary Supplements | Vitamins (C and D) |
|  | Behavioral modifications | Postcoital-voiding |
| Scribano, D. | Dietary Modifications | Specific diet |
| Shepherd, A. K. | Antibiotic Regimens | Low dose continuous antibiotic prophylaxis |
|  | Behavioral modifications | Avoiding spermicide-based contraceptives (diaphragm use, condoms etc.) |
|  | Behavioral modifications | Avoiding spermicide-based contraceptives (diaphragm use, condoms etc.) |
|  | Behavioral modifications | Limiting use of spermicides |
|  | Dietary Supplements | Cranberry products |
|  | Behavioral modifications | Avoiding postponing urination |
|  | Behavioral modifications | Avoiding skin allergens (soaps, vaginals creams, bubble baths, hot tubs etc.) |
|  | Pharmaceuticals | Anti-infective: Methenamine Hippurate |
|  | Behavioral modifications | Wear cotton underwear |
|  | Antibiotic Regimens | Postcoital antibiotic prophylaxis |
|  | Dietary Supplements | Probiotics (different Lactobacilli) |
|  | Behavioral modifications | Avoiding skin allergens (soaps, vaginals creams, bubble baths, hot tubs etc.) |
|  | Behavioral modifications | Postcoital-voiding |
|  | Behavioral modifications | Proper wiping patterns |
| Sihra, N. | Behavioral modifications | Avoiding anal intercourse |
|  | Behavioral modifications | Avoiding spermicide-based contraceptives (diaphragm use, condoms etc.) |
|  | Behavioral modifications | Avoiding skin allergens (soaps, vaginals creams, bubble baths, hot tubs etc.) |
|  | Behavioral modifications | Avoiding sexual intercourse |
|  | Behavioral modifications | Limiting use of spermicides |
|  | Complementary Approaches | Chinese herbal medicines |
|  | Behavioral modifications | Wear cotton underwear |
|  | Dietary Supplements | Cranberry products |
|  | Dietary Supplements | D-mannose |
|  | Dietary Modifications | Increased hydration |
|  | Pharmaceuticals | Immunostimulants (vaccines) |
|  | Medical procedures | Intravesical installations |
|  | Pharmaceuticals | Anti-infective: Methenamine Hippurate |
|  | Pharmaceuticals | NSAID |
|  | Dietary Supplements | Probiotics (different Lactobacilli) |
|  | Behavioral modifications | Avoiding tampons/prolonged use of pads |
|  | Pharmaceuticals | Immunostimulants (vaccines) |
|  | Dietary Supplements | Vitamins (C and D) |
|  | Behavioral modifications | Avoiding postponing urination |
|  | Behavioral modifications | Proper wiping patterns |
| Sihra, N. | Behavioral modifications | Limiting use of spermicides |
|  | Antibiotic Regimens | Low dose continuous antibiotic prophylaxis |
|  | Dietary Supplements | Cranberry products |
|  | Dietary Supplements | D-mannose |
|  | Medical procedures | Fecal transplantation |
|  | Pharmaceuticals | Immunostimulants (vaccines) |
|  | Antibiotic Regimens | Self-start antibiotics |
|  | Medical procedures | Intravesical installations |
|  | Pharmaceuticals | Anti-infective: Methenamine Hippurate |
|  | Medical procedures | Fractional CO2 laser and distal urethral transposition |
|  | Antibiotic Regimens | Postcoital antibiotic prophylaxis |
|  | Dietary Supplements | Probiotics (different Lactobacilli) |
|  | Medical procedures | Urethral dilation |
|  | Pharmaceuticals | Immunostimulants (vaccines) |
| Silverman, J. A. | Dietary Supplements | Cranberry products |
|  | Antibiotic Regimens | Low dose continuous antibiotic prophylaxis |
|  | Dietary Supplements | Anti-adhesive therapeutics: Pillicides and mannosides |
|  | Pharmaceuticals | Immunostimulants (vaccines) |
| Smith, A. L. | Antibiotic Regimens | Postcoital antibiotic prophylaxis |
|  | Complementary Approaches | Acupuncture |
|  | Dietary Modifications | Increased hydration |
|  | Behavioral modifications | Avoiding postponing urination |
|  | Behavioral modifications | Avoiding sexual intercourse |
|  | Dietary Supplements | Cranberry products |
|  | Dietary Supplements | D-mannose |
|  | Complementary Approaches | Chinese herbal medicines |
|  | Medical procedures | Intravesical installations |
|  | Dietary Supplements | Probiotics (different Lactobacilli) |
|  | Antibiotic Regimens | Low dose continuous antibiotic prophylaxis |
|  | Pharmaceuticals | Anti-infective: Methenamine Hippurate |
|  | Pharmaceuticals | Immunostimulants (vaccines) |
|  | Behavioral modifications | Postcoital-voiding |
| Sosland | Dietary Supplements | Cranberry products |
|  | Dietary Supplements | D-mannose |
|  | Dietary Modifications | Increased hydration |
|  | Medical procedures | Intravesical installations |
|  | Dietary Supplements | Probiotics (different Lactobacilli) |
|  | Pharmaceuticals | Anti-infective: Methenamine Hippurate |
| Stair, S. L | Dietary Supplements | Cranberry products |
|  | Dietary Supplements | D-mannose |
|  | Dietary Modifications | Increased hydration |
|  | Pharmaceuticals | Anti-infective: Methenamine Hippurate |
| Taich, L. | Dietary Supplements | Cranberry products |
|  | Antibiotic Regimens | Low dose continuous antibiotic prophylaxis |
| Tamadonfar, K. O. | Dietary Supplements | Glycolipids/galactocides |
|  | Dietary Supplements | Glycolipids/galactocides |
|  | Pharmaceuticals | Immunostimulants (vaccines) |
| Vahlensieck, W. | Dietary Supplements | Other acidifying agents |
|  | Antibiotic Regimens | Low dose continuous antibiotic prophylaxis |
|  | Dietary Supplements | Cranberry products |
|  | Dietary Supplements | D-mannose |
|  | Dietary Modifications | Increased hydration |
|  | Medical procedures | Intravesical installations |
|  | Pharmaceuticals | Immunostimulants (vaccines) |
|  | Dietary Supplements | Probiotics (different Lactobacilli) |
|  | Pharmaceuticals | Anti-infective: Methenamine Hippurate |
|  | Complementary Approaches | Phytoterapeutics |
|  | Antibiotic Regimens | Postcoital antibiotic prophylaxis |
|  | Behavioral modifications | Avoiding postponing urination |
|  | Behavioral modifications | Proper wiping patterns |
| Van Wietmarschen | Dietary Supplements | Other acidifying agents |
|  | Complementary Approaches | Phytoterapeutics |
|  | Complementary Approaches | Chinese herbal medicines |
|  | Dietary Supplements | Cranberry products |
| Vecchio | Behavioral modifications | Limiting use of spermicides |
|  | Dietary Supplements | Cranberry products |
|  | Behavioral modifications | Avoiding postponing urination |
|  | Behavioral modifications | Proper wiping patterns |
|  | Dietary Modifications | Increased hydration |
|  | Antibiotic Regimens | Low dose continuous antibiotic prophylaxis |
|  | Antibiotic Regimens | Postcoital antibiotic prophylaxis |
|  | Dietary Supplements | Probiotics (different Lactobacilli) |
| Vedanayagam, M. | Medical procedures | Intravesical installations |
| Wagenlehner, F. | Antibiotic Regimens | Low dose continuous antibiotic prophylaxis |
|  | Dietary Supplements | Cranberry products |
|  | Dietary Supplements | D-mannose |
|  | Complementary Approaches | Phytoterapeutics |
|  | Pharmaceuticals | Immunostimulants (vaccines) |
|  | Dietary Modifications | Increased hydration |
|  | Pharmaceuticals | Anti-infective: Methenamine Hippurate |
|  | Antibiotic Regimens | Postcoital antibiotic prophylaxis |
|  | Dietary Supplements | Probiotics (different Lactobacilli) |
|  | Antibiotic Regimens | Self-start antibiotics |
|  | Behavioral modifications | Behavioral modifications (non specified) |
| Wasson. | Behavioral modifications | Limiting use of spermicides |
|  | Dietary Supplements | Cranberry products |
|  | Antibiotic Regimens | Low dose continuous antibiotic prophylaxis |
|  | Pharmaceuticals | Anti-infective: Methenamine Hippurate |
|  | Behavioral modifications | Avoiding spermicide-based contraceptives (diaphragm use, condoms etc.) |
|  | Antibiotic Regimens | Postcoital antibiotic prophylaxis |
|  | Pharmaceuticals | Immunostimulants (vaccines) |
| Yang, B. | Dietary Supplements | Cranberry products |
|  | Dietary Supplements | D-mannose |
|  | Pharmaceuticals | Immunostimulants (vaccines) |
|  | Pharmaceuticals | Immunostimulants (vaccines) |
|  | Medical procedures | Intravesical installations |
|  | Pharmaceuticals | Anti-infective: Methenamine Hippurate |
|  | Dietary Supplements | Probiotics (different Lactobacilli) |
| Zak, D. | Antibiotic Regimens | Low dose continuous antibiotic prophylaxis |
|  | Behavioral modifications | Avoiding spermicide-based contraceptives (diaphragm use, condoms etc.) |
|  | Dietary Supplements | Cranberry products |
|  | Dietary Modifications | Increased hydration |
|  | Antibiotic Regimens | Self-start antibiotics |
|  | Antibiotic Regimens | Postcoital antibiotic prophylaxis |
|  | Dietary Supplements | Probiotics (different Lactobacilli) |
|  | Behavioral modifications | Postcoital-voiding |
| Zare, M. | Others | Bacteriophages |
|  | Antibiotic Regimens | Low dose continuous antibiotic prophylaxis |
|  | Dietary Supplements | Cranberry products |
|  | Behavioral modifications | Avoiding skin allergens (soaps, vaginals creams, bubble baths, hot tubs etc.) |
|  | Dietary Supplements | D-mannose |
|  | Medical procedures | Fecal transplantation |
|  | Medical procedures | Intravesical installations |
|  | Behavioral modifications | Avoiding hypothermia |
|  | Pharmaceuticals | Immunostimulants (vaccines) |
|  | Pharmaceuticals | Immunostimulants (vaccines) |
|  | Dietary Modifications | Increased hydration |
|  | Pharmaceuticals | Anti-infective: Methenamine Hippurate |
|  | Complementary Approaches | Phytoterapeutics |
|  | Antibiotic Regimens | Postcoital antibiotic prophylaxis |
|  | Dietary Supplements | Probiotics (different Lactobacilli) |
|  | Behavioral modifications | Avoiding anal intercourse |
